# Modulation of response inhibition with event-related atVNS showed EEG but no behavioral effects

**DOI:** 10.1038/s41598-025-24491-w

**Published:** 2025-11-19

**Authors:** Leonie F. Becker, Gesine M. Sallandt, Eva Rosolowsky, Sophie Hetzel, Christian Frings, Tobias Bäumer, Moritz Mückschel, Christian Beste, Alexander Münchau

**Affiliations:** 1 Institute of Systems Motor Science, University of Lübeck, Lübeck, Germany; 2 Department of Pediatrics, University Hospital Medical Center Schleswig-Holstein, Campus Lübeck, Lübeck, Germany; 3 Department of Radiology, Sahlgrenska University Hospital, Gothenburg, Sweden; 4 Cognitive Psychology, Institute of Psychology, University of Trier, Trier, Germany; 5 Cognitive Neurophysiology, Department of Child and Adolescent Psychiatry, Faculty of Medicine, TU Dresden, Schubertstraße 42, 01307 Dresden, Germany; 6 Faculty of Medicine, University Neuropsychology Center, TU Dresden, Dresden, Germany; 7 Cognitive Psychology, Faculty of Psychology, Shandong Normal University, Jinan, China

**Keywords:** AtVNS, TEC, Response inhibition, Noradrenaline, Human behaviour, Cognitive neuroscience

## Abstract

Response inhibition can be explained within the theory of event coding. Event file binding and reconfiguration have previously been studied in relation to various neurotransmitters. We aimed to activate the noradrenergic system through auricular transcutaneous vagus nerve stimulation (atVNS) to study effects on response inhibition. Forty healthy participants completed a Go/Nogo task under verum and sham phasic event-related atVNS stimulation with concomitant EEG measurement. The Go/Nogo task was designed to assess response inhibition with feature overlap between Go and Nogo trials requiring reconfiguration of event files. In overlapping Go trials, reaction times increased, and accuracy decreased compared to non-overlapping trials. False alarm rates were higher in overlapping Nogo trials. There were no influences of atVNS on behavioral data. Using multivariate pattern analysis, EEG data showed sustained and robust differences in classification between verum and sham stimulation across trial types. Off-diagonal activity was consistently observed in time generalization matrices between 100 and 900ms for overlapping Go and Nogo conditions, as well as for non-overlapping Go conditions and until approximately 750ms for non-overlapping Nogo conditions. There were no trial-specific effects of phasic atVNS, it affected all trials in the verum condition. In conclusion, this study demonstrates that short-lasting, event-related atVNS has an effect on EEG activities, but not on behavioral outcomes during a Go/Nogo task that requires event-file reconfiguration. As EEG changes were detected also in non-stimulated trials, there may be effects of the short-duration atVNS outlasting the period of stimulation.

**Supplementary Information:**

The online version contains supplementary material available at 10.1038/s41598-025-24491-w.

## Introduction

Response inhibition plays an important role in our ability to select the most suitable behavior. The theory of event coding (TEC)^1^ offers a theoretical framework for understanding these response inhibition processes. According to TEC, presenting a stimulus that requires a specific response creates an association between the stimulus and the response^1^. These associations are established in ‘event files,’ which represent stimuli and actions by their constituting features^1–3^. When a feature of a previously integrated stimulus is shown again, the event file can be reactivated, triggering the associated response. However, problems can arise if a nearly identical stimulus input triggers opposing actions, because the previously established stimulus-response bindings are only partially fulfilled. This violation of expectations interferes with the associations and requires the reconfiguration of the event file^4–7^.

Several studies have explored the neurobiochemical basis of event file coding processes. For example, an influence of the dopaminergic system was shown pharmacologically^8^ or in patients with a known involvement of the dopaminergic system^9^. GABA seems to play a minor role in event file binding and has different effects as a function of anatomical localization. Thus, striatal GABA levels do not modulate event file binding, while GABA in the anterior cingulate cortex appears to enhance event file binding, but only in participants who have not yet acquired sufficient task experience^10^. When healthy individuals were administered Methylphenidate, which increases levels of catecholamines such as dopamine and noradrenaline, they exhibited fewer false alarms in a response inhibition task that involved response overlap^11^. This effect was particularly pronounced if they were able to voluntarily regulate theta band activity^11^. These findings suggest that catecholamine levels may modulate binding. A possible influence of the noradrenergic (NE) system on event files is underlined by different theoretical considerations. According to Verguts and Notebaert^12^, the binding of stimulus and response features into event files may be facilitated by phasic increases of NE released in the locus coeruleus. Conflict signals may prompt an arousal response in this system, which may act as a reinforcement signal that boosts currently active task-relevant representations^12^. Therefore, arousal prompted by the conflict may boost reconfiguration processes in event files, i.e., decrease hyper-binding^12^. Furthermore, NE influences gain control that is a general principle in the central nervous system at sensory, cognitive^13^, and motor levels^14^. Gain control may also be related to binding processes^11,15^. The coding of event files may be influenced by the interaction of systems that promote cognitive persistence, like focusing on relevant information, and systems that promote cognitive flexibility, like considering a wider range of possibilities^16,17^. The modulation between these systems could be achieved through gain control^18^, which in turn is modulated by the NE system^19^. According to the adaptive gain theory, phasic NE responses facilitate task-relevant decision-making processes in prefrontal cortices^19^. Since high gain control is associated with less interference^20^, it likely increases cognitive persistence and thus enhances reconfiguration processes in event files^15,21^.

Previous studies suggest that auricular transcutaneous vagus nerve stimulation (atVNS) may influence the NE and GABA systems^22^. Very short-lasting atVNS was shown to enhance rapid phasic neural activity in the NE locus coeruleus of rats^23^. In humans, short-term atVNS of 3,4 s could influence pupil size^24,25^ and alpha power in EEG^24^, both indirect signs of locus coeruleus activity^24,26^. Wienke et al. showed short-lasting atVNS to enhance accuracy and increase frontal-midline theta and alpha power in EEG as well as pupil dilation in a Stroop task^27^. In contrast, Villani et al. demonstrated that three-second event-related atVNS increases reaction times and decreases pupil dilation in an auditory oddball task, although the latter was dependent on baseline pupil size^28^. AtVNS was also found to significantly enhance GABA activity, according to a study that used short-interval intracortical inhibition, a transcranial magnetic stimulation paradigm sensitive to GABA activity^29^. It also modulated the negative compatibility effect in a subliminal priming task, which is known to be highly correlated to GABA concentrations^30^. It is therefore plausible to assume that applying atVNS allows to investigate causal relationships between neurotransmitter systems^22,31^ and dependent cognitive functions. Since gain control is conceptualized in the adaptive gain theory as a mechanism that works transiently, we assume that especially the transient (event-related) modulation of neurophysiological systems (in particular the NE system) should modulate cognitive functions. Therefore, we used an event-related atVNS approach similar to Villani et al.^28^ to transiently modulate neurobiological processes underlying event-file processing.

Our goal was to investigate the influence of atVNS on response inhibition. As outlined above, we hypothesized that it may influence the coding of event files by activating the NE system, which may influence the coding of event files by boosting reconfiguration processes and influencing gain control. We used a task used in previous studies to investigate response inhibition in the context of TEC^5^. This Go/Nogo task comprises conditions with and without feature overlap between Go and Nogo trials. This means that event file reconfiguration is needed for Go and Nogo trials with feature overlap^5,11^, although the extent may differ^32^. In Nogo trials, additional inhibitory processes play a role^32^. Previous research has demonstrated that response inhibition is impaired when stimulus features overlap between Go and NoGo trials and false alarm rates are increased when NoGo stimulus features overlap with Go stimulus features^5^. We anticipated that due to better reconfiguration of event-files, participants would exhibit higher accuracy and reduced false alarms when Go/NoGo stimulus features overlap during active atVNS stimulation as compared to sham stimulation. In addition to behavioral measures, event-file binding can be examined neurophysiologically using EEG allowing a better understanding of the underlying processes^6^. When analyzing EEG signals during atVNS stimulation, it is crucial to note that an event file is a representational structure^1,33^. Using multivariate pattern analysis (MVPA) it is possible to decode the representational differences between conditions in neurophysiological data^34^, because MVPA provides information about the temporal stability of these representations^35^. Recent studies have shown that MVPA can capture sustained representations between 100 and 750 ms after stimulus representation in the response inhibition task used in this study^11,32^. It has been suggested that this time period reflects the gradual activation followed by the deactivation of event files during response inhibition^32^. If atVNS has an impact on response inhibition at a neurophysiological level, a classifier would detect differences between verum and sham conditions. Additionally, we hypothesize that phasic, i.e. trial-related atVNS would only or predominantly affect trials in which active stimulation is applied but not or to a lesser degree trials where stimulation is paused, because previous studies showed short-lasting effects of stimulation^24,25,27,28,36^.

## Methods

### Participants

The study investigated *N* = 44 healthy participants between the ages of 18 and 30 who had no current psychiatric or neurological diseases, stable medication intake, and were right-handed. Three participants were taking oral contraceptives, one person each was taking levothyroxine, candesartan, ramipril, mesalazine, dupilumab, cetirizine or vitamin B12. One participant was taking candesartan, hydrochlorothiazide and lercanidipine. Another participant was taking iron and monk’s pepper. Participants with red-green color blindness were excluded. All subjects provided written informed consent. The study was approved by the ethics committee of the University of Lübeck and was conducted in accordance with the Declaration of Helsinki. Three subjects were excluded from the analysis due to technical issues with the measurements. One participant exhibited tics during the measurement and was therefore excluded. Data from a total of 40 subjects were analyzed, comprising 22 females and 18 males with an average age of 23.2 ± 2.8 years.

### Task

**Fig. 1:**
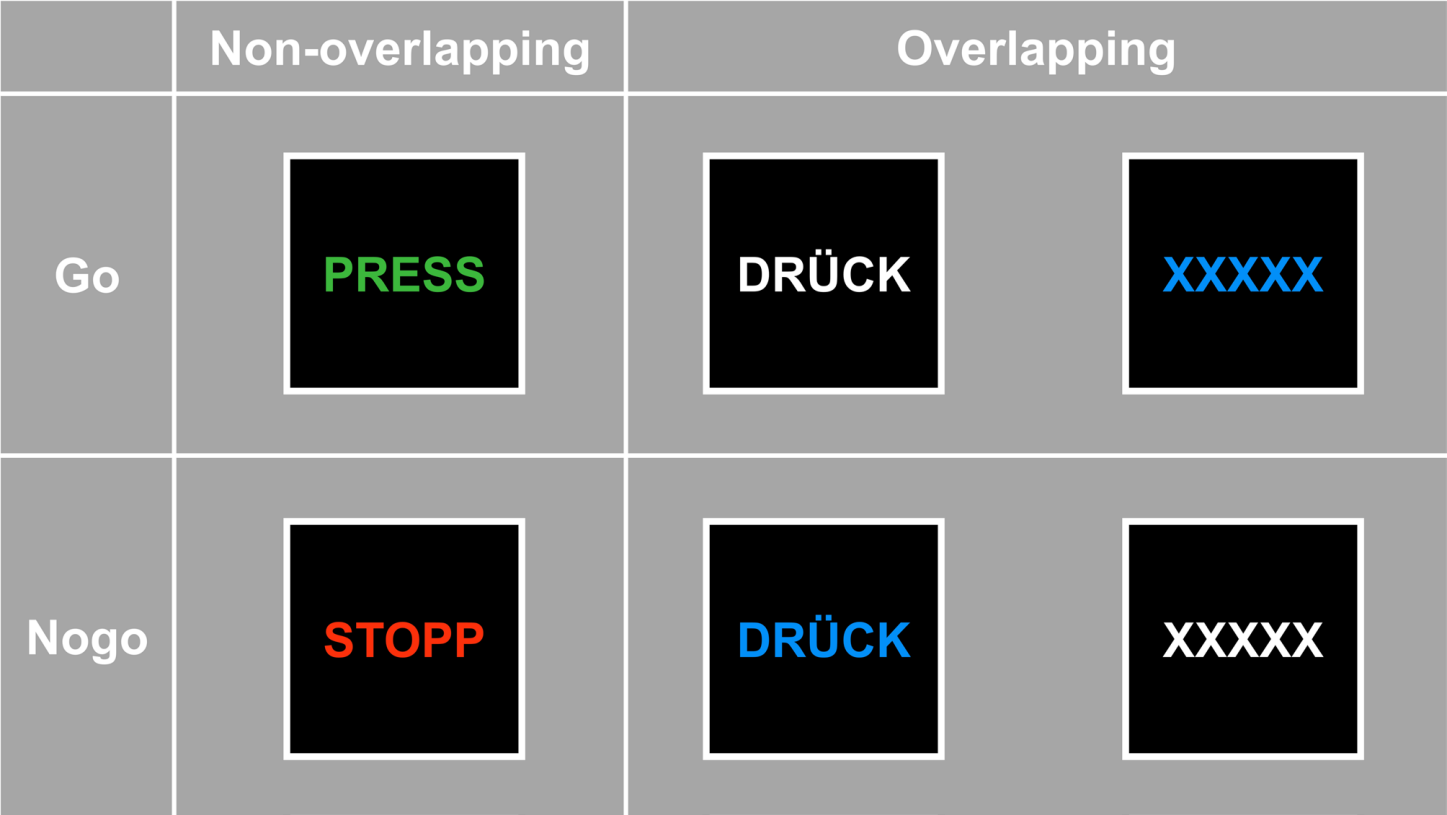
Experimental conditions used to induce overlaps in event files^5^. For Go trials, the word ‘PRESS’ in green color, ‘DRÜCK’ in white color or the letters ‘XXXXX’ in blue color were presented. For the Nogo condition, the word ‘STOPP’ in red color, the word ‘DRÜCK’ in blue color or the letters ‘XXXXX’ in white color were presented. The stimulus features displayed on the right showed feature overlap between Go and Nogo trials. The illustration is based on the illustration by Chmielewski and Beste^5^.

An adjusted version of the ‘TEC Go/Nogo’ task^5^ was used to investigate the reconfiguration processes of stimulus-response reactions (event files). The task consisted of Go and Nogo trials with and without stimulus overlap. There were two ‘overlapping’ and one ‘non-overlapping’ trial types for both Go and Nogo condition. The non-overlapping Go trials displayed a green ‘PRESS’ and the non-overlapping Nogo trials displayed a red ‘STOP’. In contrast, the ‘overlapping’ Go trials presented either a white ‘DRÜCK’ or a blue ‘XXXXX’ as stimuli, while the ‘overlapping’ Nogo trials comprised a blue ‘DRÜCK’ or a white ‘XXXXX’ (see Fig. 1). A ratio of five Go trials to three Nogo trials was selected, and the trials were presented in a randomized order.

**Fig. 2:**
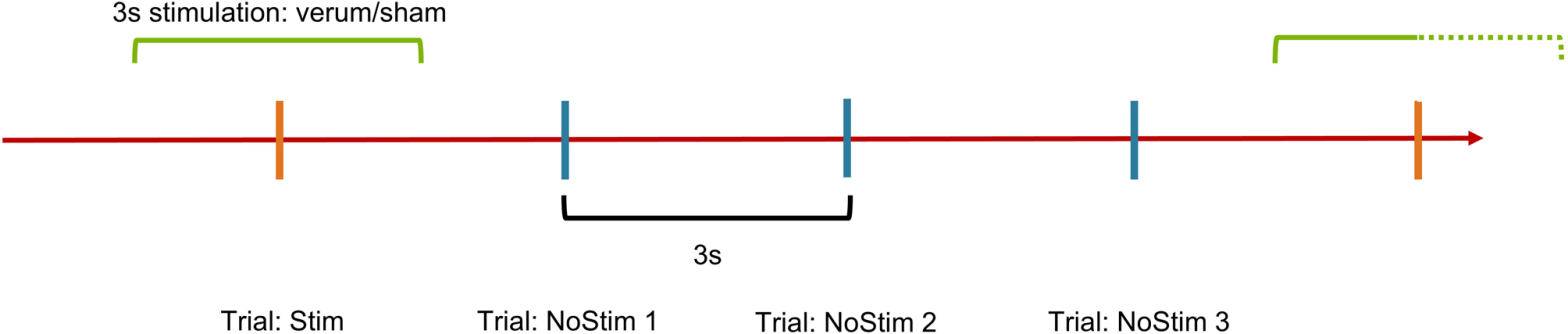
Visual timeline of stimulation in regard to the task. Event-related atVNS was applied by stimulating 1.5 s before and after the first of four trials, followed by three unstimulated trials (NoStim 1/NoStim 2/NoStim 3). The resultant sequence was as follows: a 3-second stimulation phase, succeeded by a 9-second pause.

Each trial lasted 1.5 s and consisted of a 0.5-second presentation of the stimulus. The intertrial interval was fixed at 1.5 s. Event-related atVNS was applied by stimulating 1.5 s before and after the first of four trials, followed by three unstimulated trials. This resulted in a 3-second stimulation phase followed by a 9-second pause (Fig. 2). The task consisted of 6 blocks à 14 min with breaks between blocks.

### AtVNS

For event-related atVNS, we used tVNS^®^ R (tVNS Health GmbH, Grünwald, Germany). Participants underwent two measurements on separate days with an interval of at least 1 week: one with verum stimulation on the left cymbae conchae^37,38^ and one with sham stimulation on the left earlobe^24,25,28^. The order of sessions was counterbalanced between the subjects. Before the task, the stimulation intensity was individually set for each participant and session, adapted from Fischer et al.^39^ and Ventura-Bort et al.^40^. To determine the stimulation intensity just below the pain threshold, participants were asked to rate their experience on a 0–10 visual analog scale, while the stimulation intensity was increased in a stepwise approach, starting at 100 µA. The scale ranged from no sensation (0) to light tingling (3), strong tingling (6), to painful (10). Once the participant rated a 9 for the ‘tingling’ sensation, the intensity was gradually decreased in 100 µA steps until a subjective sensation of 6 or below was experienced. This process was repeated twice, and the final stimulation intensity used for the experimental procedure was calculated as the average of the four intensities rated as 8 (two from the increasing series and two from the decreasing series). Verum stimulation was performed with a mean current of 1343.75 µA (± 475.8 µA), while sham stimulation was performed with 1526.25 µA (± 610.6 µA). The pulse width was 200–300 µs, frequency was 25 Hz, stimulation duration was 3 s.

### Behavioral analysis

Accuracy and reaction time were assessed for Go trials, and false alarm rate was assessed for Nogo trials, separately for verum and sham, as well as for stimulated and non-stimulated trials. Normal distribution was tested using the Shapiro-Wilk test. To evaluate the effects of verum and sham stimulation, we conducted repeated measures ANOVAs using within factors session (verum/sham), stimulation (stimulated/first non-stimulated, second non-stimulated, and third non-stimulated trials), and overlap (overlapping/non-overlapping). To compare differences between overlapping and non-overlapping trials, accuracy, reaction times, and false alarm rates were analyzed using the Student’s t-test and Wilcoxon statistic, depending on the normal distribution of variables.

### EEG

EEG was recorded using 60 Ag/AgCl electrodes (Custom 64Ch-BrainCap, BrainProducts Inc., Gliching, Germany) in equidistant positions and the Brain Vision Recorder software (Brain Products Inc., Gilching, Germany). We used a sampling rate of 500 Hz and impedances were kept below 5kΩ, if possible. Automated EEG preprocessing was done similar to previous studies^41^, using automagic^42^ and EEGLAB^43^ on Matlab 2019a (The MathWorks Corp., Natick, Massachusetts, USA). First, data was down-sampled to 256 Hz. The EEG data was re-referenced to an average reference after removing flat channels. Then, the PREP preprocessing pipeline^44^ and the EEGLAB clean_rawdata() pipeline were utilized. PREP eliminates line noise at 50 Hz using a multitaper algorithm, removes contaminations by bad channels and applies a robust average reference afterwards. Clean_rawdata() detrends the EEG data with a FIR highpass filter of 0.5 Hz (order 1286, stop-band attenuation 80 dB, transitionIIR band 0.25–0.75 Hz) and removes flat-line, noise, and outlier. Epochs displaying abnormally strong power (> 15 standard deviations relative to calibration data) were reconstructed using Artifact Subspace Reconstruction (ASR; burst criterion: 15;^45^. If reconstruction was not possible, time windows were excluded. After that, we used a low-pass filter with a cutoff frequency of 40 Hz (sinc FIR filter; order: 86;^46^. With a subtraction method^47^, EOG artifacts were eliminated. An independent component analysis (ICA) was conducted using the EEGLAB runica() function implementing the infomax ICA algorithm^48^. The independent components were classified using the Multiple Artifact Rejection Algorithm (MARA;^49,50^, remaining eye, muscle and loose electrode artifacts were removed. Cardiac artifacts were detected and removed using ICLabel^51^. Using a spherical method, all automatically removed channels were interpolated. After EEG data preparation, we generated segments for the overlapping Go and Nogo condition and for the non-overlapping Go and Nogo condition separately for stimulated and non-stimulated trials. Segments started 2000 ms prior to stimulus presentation and ended 2000 ms thereafter (segment length: 4000 ms). An automated artifact rejection procedure was used to eliminate segments with an amplitude above 200µV or below − 200 µV and the activity below 0.5 µV in a 100-msec interval. In 39 out of 40 participants, this artifact rejection led to the exclusion of up to 1% of trials. There was only one participant where about 20% of trials had to be rejected. Subsequently, we conducted a baseline correction in a time window of -200–0 ms prior to stimulus presentation.

### Multivariate pattern analysis (MVPA)

MVPA was performed on the segmented single-trial EEG data using the MVPA light toolbox^52^. This approach was chosen because atVNS and the activity of the locus coeruleus are thought to influence brain activity in various networks, rather than at a single localization. A support vector machine (SVM) classifier was trained to differentiate between verum and sham, as well as stimulated and non-stimulated trials, for each condition. The EEG amplitudes recorded at individual electrode channels in the time frame of 500ms before and 1500ms after the stimulus were used as classification features. The data was z-scored by scaling and centering it at 0 to prevent biased classification. To mitigate class imbalances in the number of trials, undersampling was performed on the training set by randomly selecting and removing trials from the majority class until class sizes were balanced. The numbers of trials used are reported in supplementary information table S1 and S2. To further improve the signal-to-noise ratio^52–54^, we employed an average sampling approach: Within each class, samples were partitioned into groups of five, and the samples in a given group were substituted with the group’s mean value. We applied k-fold cross-validation method with k = 5. Each dataset was randomly divided into 5 folds. One fold was used for testing while the remaining four folds were used for training, and the process was repeated until each fold has served as the test set once. Cluster-based permutation tests were performed to determine the time points at which the AUC significantly differed from the chance level (0.5), using Wilcoxon tests with a threshold of *p* = 0.01. Clustering was applied along the time axis. To create a null distribution, data was permuted with 2000 random draws by swapping the AUC value and its null value. The sum of all Wilcoxon values within time points was used to calculate the statistical values of the cluster-based permutation tests. To evaluate differential effects of stimulation on overlapping and non-overlapping conditions, classification accuracy (AUC) was compared between conditions, separately for Go and Nogo trials and stimulated and non-stimulated trials using FDR-corrected t-tests. To evaluate the stability of the representations, we computed temporal generalization matrices. This process identified clusters of contiguous time samples that remain significant after random permutation. The model was trained on one time point, and its discrimination performance was tested on the remaining time points. This was repeated for each time point. If training and testing were done at the same time point, it is referred to as ‘diagonal decoding’; if they occurred at different time points, it was referred to as ‘off-diagonal decoding’.

### Source localization analysis

To analyze the significant differential influences of verum and sham stimulation, we performed source localization analyses for time windows, in which differences in classification performance (verum/sham) between overlapping and non-overlapping were previously shown. We used standardized low-resolution brain electromagnetic tomography (sLORETA;^55^, as in previous studies from our group^5,56^. The sLORETA software divides the intracerebral volume into 6239 voxels at 5 mm spatial resolution. The standardized current density at each voxel is computed in a realistic head model using the MNI152 template^57^. Voxel-based sLORETA images were compared between verum and sham stimulation using sLORETA-built-in voxel-wise randomization tests with 2000 permutations, based on statistical nonparametric mapping. Voxels that showed significant differences between the contrasted conditions (corrected for multiple comparisons, *p* < 0.050) were located in the Montreal Neurological Institute (MNI) brain.

## Results

### Behavioral

For the behavioral data, to evaluate event-related differences of stimulation on accuracy, repeated measures ANOVA was conducted with within factors session (verum/sham), stimulation (stimulated/first non-stimulated, second non-stimulated and third non-stimulated trials) and overlap (overlapping, non-overlapping trials). No significant interaction was found, but there was an effect of overlap (F(1,39) = 20.625, *p* < 0.001, partial η^2^ = 0.346) (see Table 1). Accuracy was significantly higher in all non-overlapping Go trials compared to overlapping Go trials (see Table 2).

**Table 1:** Statistical analysis for behavioral data.

	Hits	RT	FA
Session	F(1,39) = 0.072, *p* = 0.790, partial η^2^=0.002	F(1,39) = 0.222, *p* = 0.640, partial η^2^=0.006	F(1,39) = 1.184, *P* = 0.283, partial η^2^=0.029
Overlap	F(1,39) = 20.625, *p* < 0.001, partial η^2^=0.346	F(1,39) = 118.289, *p* < 0.001, partial η^2^=0.752	F(1,39) = 53.152, *p* < 0.001, partial η^2^=0.577
Stimulation	F(3,117) = 2.038, *p* = 0.112, partial η^2^=0.050	F(3,117) = 2.864, *p* = 0.040, partial η^2^=0.068	F(3,117) = 0.091, *p* = 0.965, partial η^2^=0.002
Session*Overlap	F(1,39) = 0.0001, *p* = 0.992, partial η^2^=0.000003	F(1,39) = 0.012, *p* = 0.913, partial η^2^=0.0003	F(1,39) = 0.886, *p* = 0.352, partial η^2^=0.022
Session*Stimulation	F(3,117) = 1.985, *p* = 0.120, partial η^2^=0.048	F(3,117) = 0.861, *p* = 0.464, partial η^2^=0.022	F(3,117) = 0.804, *p* = 0.494, partial η^2^=0.020
Overlap*Stimulation	F(3,117) = 0.141, *p* = 0.936, partial η^2^=0.004	F(3,117) = 0.753, *p* = 0.523, partial η^2^=0.019	F(3,117) = 0.161, *p* = 0.922, partial η^2^=0.004
Overlap*Stimulation*Session	F(3,117) = 1.017, *p* = 0.388, partial η^2^=0.025	F(3,117) = 0132, *p* = 0.941, partial η^2^=0.003	F(3,117) = 0.738, *p* = 0.532, partial η^2^=0.019

Repeated measures ANOVAs for accuracy and reaction times (RT) in Go trials as well as for false alarm rates (FA) in Nogo trials with factors session (verum/sham), stimulation (stimulated/first non-stimulated/second non-stimulated/third non-stimulated trials) and overlap (overlapping/non-overlapping trials).

**Table 2:** Behavioral results and statistical analysis.

Session	Verum								Sham							
Stimulation	Stim		NoStim1		NoStim2		NoStim3		Stim		NoStim1		NoStim2		NoStim3	
Trial	o	n	o	n	o	n	o	n	o	n	o	n	o	n	o	n
Accuracy	0.93	0.98	0.93	0.97	0.93	0.97	0.93	0.97	0.93	0.97	0.93	0.97	0.94	0.98	0.93	0.98
Statistic of accuracy	W = 21.5		W = 102.0		W = 59.0		W = 43.0		W = 31.0		W = 69.0		W = 21.0		W = 60.0	
	z=− 4.628		z=− 3.489		z=− 4.413		z=− 4.654		z=− 4.745		z=− 4.480		z=− 4.986		z=− 4.177	
	*p* < 0.001		*p* < 0.001		*p* < 0.001		*p* < 0.001		*p* < 0.001		*p* < 0.001		*p* < 0.001		*p* < 0.001	
RT	565.9	528.7	569.5	531.5	567.2	527.9	568.5	528.7	571.4	532.7	572.1	534.1	569.0	530.7	570.5	529.3
Statistic of RT	t = 9.384		t = 8.482		t = 8.958		t = 8.126		t = 8.070		t = 9.022		t = 8.224		t = 8.424	
	df = 39		df = 39		df = 39		df = 39		df = 39		df = 39		df = 39		df = 39	
	*p* < 0.001		*p* < 0.001		*p* < 0.001		*p* < 0.001		*p* < 0.001		*p* < 0.001		*p* < 0.001		*p* < 0.001	
FA	0.139	0.006	0.142	0.004	0.139	0.005	0.148	0.005	0.132	0.005	0.131	0.004	0.133	0.005	0.128	0.004
Statistic of FA	W = 720.0		W = 739.0		W = 780.0		W = 780.0		W = 820.0		W = 780.0		W = 814.0		W = 741.0	
	z = 5.069		z = 5.344		z = 5.442		z = 5.442		z = 5.511		z = 5.442		z = 5.430		z = 5.373	
	*p* < 0.001		*p* < 0.001		*p* < 0.001		*p* < 0.001		*p* < 0.001		*p* < 0.001		*p* < 0.001		*p* < 0.001	

Accuracy and reaction times (RT, in ms) for overlapping (o) and non-overlapping (n) Go trials, as well as false alarm rate (FA) for overlapping (o) and non-overlapping (n) Nogo trials separately for stimulated (Stim) and non-stimulated trials (NoStim1/NoStim2/NoStim3) and verum and sham stimulation. The accuracy and false alarm rates in the different trial types (overlapping and non-overlapping trials) were compared against each other using the Wilcoxon statistic. The reaction times in overlapping and non-overlapping trials were compared against each other using Student’s t-test.

Additionally, to evaluate event-related differences of stimulation on reaction time, repeated measures ANOVA was conducted with within factors session (verum/sham), stimulation (stimulated/first non-stimulated, second non-stimulated and third non-stimulated trials), and overlap (overlapping/non-overlapping trials). No significant interaction was found, but there were significant effects for overlap (F(1,39) = 118,289, *p* < 0.001, partial η^2^ = 0.752) and stimulation (F(3,117) = 2.864, *p* = 0.040, partial η^2^ = 0.068) (see Table 1). Reaction time was significantly faster in all non-overlapping Go trials compared to overlapping Go trials (see Table 2). No significant differences were found between stimulated and non-stimulated trials.

To evaluate event-related differences of stimulation on false alarm rate, repeated measures ANOVA was conducted with within factors session (verum/sham), stimulation (stimulated/first non-stimulated, second non-stimulated and third non-stimulated trials), and overlap (overlapping/non-overlapping trials). No significant interaction was found, but there was again an effect of overlap (F(1,39) = 53.152, *p* < 0.001, partial η^2^ = 0.577) (see Table 1). The false alarm rate was higher in all overlapping compared to non-overlapping Nogo trials (see Table 2).

### EEG

MVPA was conducted on the EEG data. Initially, the objective was to evaluate the differences between verum and sham stimulation. The analysis included all trial types, such as overlapping/non-overlapping Go and Nogo trials, separately for stimulated and non-stimulated trials. The analysis was limited to a time window from − 500 ms to 1500 ms relative to stimulus onset.

**Fig. 3:**
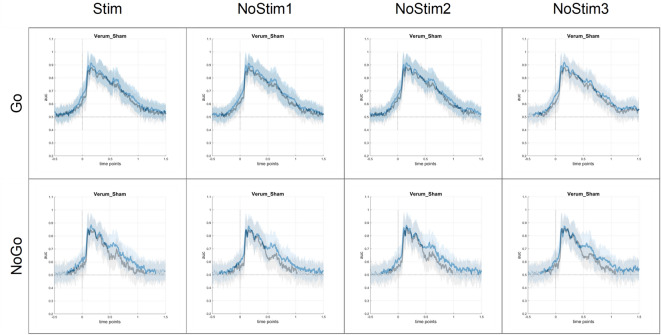
AUC curves for EEG data. The graphs display AUC curves for overlapping trials (blue) and non-overlapping trials (grey) contrasting verum and sham stimulation, separately for Go trials (upper part) and Nogo trials (lower part) as well as stimulated (Stim) and non-stimulated trials (NoStim1/NoStim2/NoStim3). The AUC value indicates the probability of the classifier distinguishing between verum and sham stimulation at a given point in time. Significant deviations from 0.5 (chance level) are indicated in bold.

Above-chance classification performance was observed between verum and sham stimulation until ~ 1100-1300 ms for Nogo trials and until 1500ms after stimulus onset for Go trials (see Fig. 3). The maximum performance, around 0.85–0.9, was observed between 100 and 200 ms after stimulus onset with a gradual decline in accuracy towards the end of the evaluated time window, for both Go and Nogo trials. Visual inspection showed no difference in classification performance of stimulated and non-stimulated trials.

To identify whether there are differences in verum stimulation depending on whether trials are stimulated or non-stimulated (first non-stimulated/ second non-stimulated/ third non-stimulated), MVPA was used to compare these different trials. The analysis was conducted separately on overlapping/non-overlapping Go and Nogo trials. There was no above-chance classification performance.

**Fig. 4:**
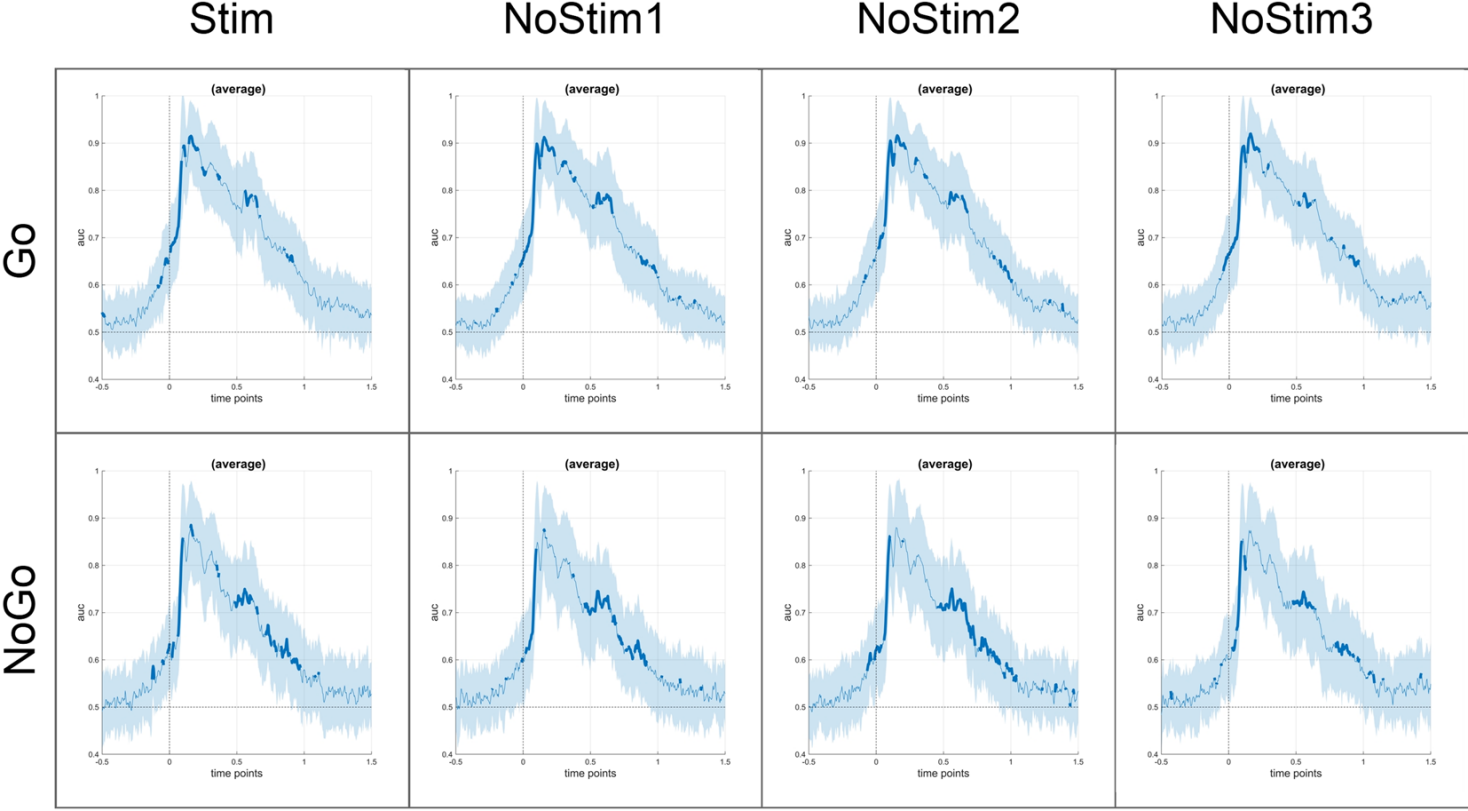
Comparison of AUC curves for EEG data. The graphs display AUC curves for overlapping trials contrasting verum and sham stimulation, separately for Go trials (upper part) and Nogo trials (lower part) as well as stimulated (Stim) and non-stimulated trials (NoStim1/NoStim2/NoStim3). Bold lines indicate significant differences between AUC curves of overlapping and non-overlapping trials. This shows the times at which different classification performances were displayed.

To evaluate differential effects of stimulation on overlapping and non-overlapping conditions, classification accuracy between verum and sham was compared between conditions, separately for Go and Nogo trials and stimulated and non-stimulated trials using FDR-corrected t-tests. Differences in classification accuracy for verum and sham between these conditions were observed in all tested conditions, mainly directly after stimulus presentation (0–200 ms) and in the time window of 500–700 ms after stimulus presentation (see Fig. 4). Visual inspection comparing stimulated vs. non-stimulated trials showed only minor differences.

**Fig. 5:**
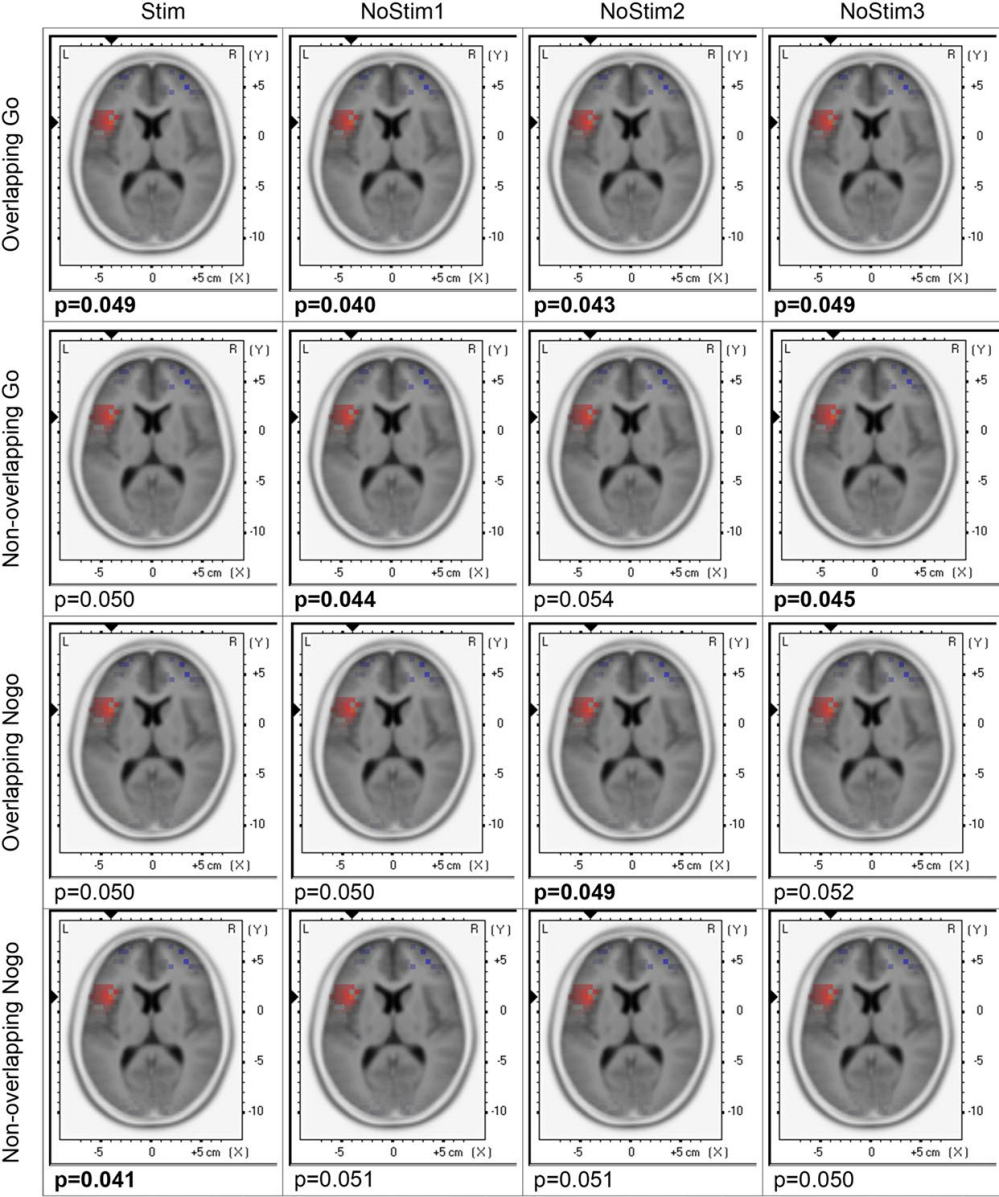
Visualization of the activity of brain regions after stimulation. sLORETA plots contrasting the effects of verum and sham stimulation within 500ms to 700ms after stimulus presentation for all trial types, such as overlapping/non-overlapping Go and Nogo trials, for both verum and sham stimulation, separately for stimulated (Stim) and non-stimulated trials (Nostim1/NoStim2/NoStim3). Significant p-values are indicated in bold. All plots demonstrate modulations in left Brodmann area 13, Insula, and sub-lobar area.

To investigate the activation pattern of verum vs. sham stimulation in the brain, sLORETA was applied. For the time windows of 0-200ms sLORETA did not show significant activation patterns. For the time window of 500-700ms, sLORETA plots, contrasting verum and sham stimulation, showed significant and trends of activation in Brodmann area 13, Insula, and sub-lobar area (see Fig. 5). Again, visual inspection did not show differences of activation between stimulated and non-stimulated trials in the verum condition.

**Fig. 6:**
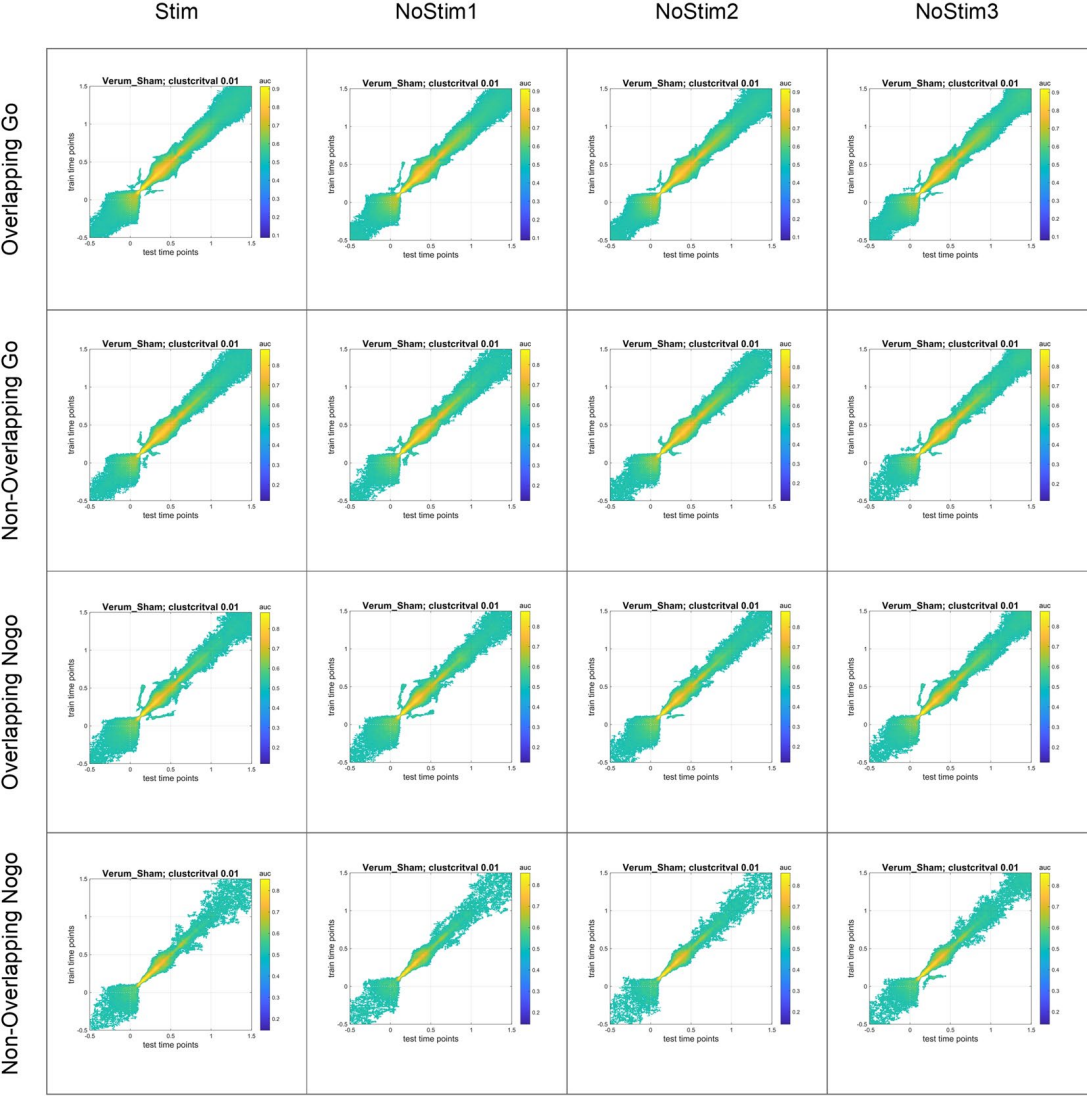
Temporal generalization matrices for EEG data. Matrices contrasting verum and sham stimulation for all trial types, such as overlapping/non-overlapping Go and Nogo trials, for both verum and sham stimulation, separately for stimulated (Stim) and non-stimulated trials (Nostim1/NoStim2/NoStim3). The yellow color indicates an above-chance generalization at time point t. The discrimination between verum and sham decodes a sharp off-diagonal pattern, indicating that the stimulation might influence the rapid decoding of the event file.

To assess the stability of the representations, we computed temporal generalization matrices. These matrices showed a significant above-chance (0.7–0.9) diagonal pattern during a time window between 100-900ms for overlapping Go and Nogo conditions, as well as for non-overlapping Go conditions. The diagonal reached peak classification performance of about 0.8 to 0.9 from 100 until 400ms (see Fig. 6). For the non-overlapping Nogo condition, the diagonal pattern was only present until approximately 750ms (see Fig. 6). Again, visual inspection did not show differences in temporal generalization between stimulated and non-stimulated trials.

## Discussion

The current study examined the influence of short-term event-related atVNS on event-file reconfiguration in a response inhibition task. To investigate this relationship, healthy participants performed a Go/Nogo task under verum and sham atVNS stimulation.

On a behavioral level, accuracy in Go conditions without feature overlap between Go and Nogo trials was higher compared to trials where features overlapped. Also, when feature overlap occurred, reaction times increased in Go trials and false alarms in Nogo trials were more frequent. These behavioral results are consistent with previous studies^5,11^ and are in line with the theory of event coding^1,33^. Overlapping trials create the necessity to reconfigure the event-file, which results in longer reaction times and more errors^5,32^.

Divergent from our hypothesis, there was no influence of atVNS on behavioral outcomes. MVPA of EEG data showed a possible classification between verum and sham stimulation, particularly during the time frame of 100-200ms after stimulus onset, regardless of the type of trial evaluated (Go or Nogo, stimulated or non-stimulated and trials with or without feature overlap). There was sustained off-diagonal activity in time generalization matrices during a time window between 100-900ms for overlapping Go and Nogo conditions, as well as for non-overlapping Go conditions and until approximately 750ms for non-overlapping Nogo conditions. Further investigations revealed that MVPA classification performance between verum and sham differed between overlapping and non-overlapping conditions, with higher accuracy in overlapping conditions between 500 and 700 ms in Nogo trials. During this time frame, sLoreta revealed distinct activity in left Brodmann area 13, insula, and sub-lobar area.

As outlined in the introduction, atVNS may influence the NE and GABA systems. While the former may play a role in the formation and reconfiguration of event files, GABA appears to have a lesser influence in this respect. Striatal GABA levels do not appear to affect event-file binding, while GABA in the anterior cingulate cortex seems to enhance event-file binding only in participants without enough task experience^10^. Our discussion will therefore focus on the influence of the NE system. There are two possible mechanisms of action. Based on the ‘adaptation by binding theory’^12^, conflict signals prompt an arousal response in the neuromodulatory locus coeruleus NE system probably boosting reconfiguration processes in event files, decreasing hyper-binding. According to adaptive gain theory, the NE system regulates gain control^19^ and facilitates task-relevant decision-making processes in prefrontal cortices^19^. Higher gain control is thought to be associated with binding processes by increasing cognitive persistence and enhancing reconfiguration processes in event files^15,20,21^.

In the EEG data, differences between verum and sham stimulation were found in the time ranges associated with activation and reconfiguration of event files^32,35^. In addition, differential effects of stimulation were shown in relation to overlapping and non-overlapping trials. A sharp diagonal pattern, as seen in the time generalization matrices, is associated with the rapid progression through successive coding stages^58^. This could be another sign of differences in the activation and reconfiguration of the event file between verum and sham stimulation. Representations between 100 and 750 ms after stimulus presentation are believed to show the gradual activation followed by deactivation of event files during response inhibition^32^. The P1 evoked potential was observed until 100-200ms after stimulus onset^32^, and is a manifestation of early stimulus categorization^32,59^. The P3 was observed in the 500-700ms time frame using this task^5,32^, and is proposed to reflect event file reconfiguration during action selection^60^. The differential effects of stimulation on overlapping and non-overlapping trials were found in regions previously associated with atVNS stimulation. According to a recent meta-analysis, verum atVNS leads to increased brain activation in the left middle insula, among other effects^61^. The results indicate that verum stimulation may affect the retrieval and rebinding of the event file, possibly through activation of the NE system. The differential effects observed in overlapping and non-overlapping conditions may suggest an enhancement of phasic activity when reconfiguration is required. However, this argument remains speculative. In addition to modulation of the NE system by atVNS, also effects on the GABAergic system need to be considered^22–24,28–30^.

If atVNS has a direct influence on binding processes by activating the NE system, we hypothesized that verum stimulation would also result in higher accuracy, faster RT, and a lower false alarm rate compared to sham stimulation. This could be attributed to improved reconfiguration of the event files in the case of overlapping conditions. Contrary to our hypothesis, our study did not demonstrate an effect on behavioral data, indicating a dissociation between behavioral and EEG data. This could be due to various factors. One argument is that the intensity of our stimulation protocol (with a mean current of 1.3 mA) was sufficient to produce EEG effects, but not sufficient to produce behavioral changes. In support of this argument, Wienke et al. used 2 mA for stimulation and demonstrated improved performance in an emotional Stroop task under verum stimulation^27^. However, it is important to note that they used a shorter stimulation time of 0.5 s. Ludwig et al. used 3 mA and 5 mA (3 s on, 15 s off) and were also able to demonstrate improved performance in an emotional memory task^62^. Conversely, Villani et al. used stimulation just below the perception threshold (mean 0.38 mA) for three seconds and demonstrated longer reaction times in an auditory oddball task^28^. Bömmer et al. also used a lower intensity (around 0.46 mA) and showed longer reaction time^63^. Additionally, these different results may not only be due to the intensity, but also to the pulse width and frequency, as Hulsey et al. demonstrated^23^. Further studies comparing different stimulation protocols are needed, as they may have different effects.

Unlike previous studies^27,28,62^, which demonstrated effects, we employed a different task. It is possible that our healthy participants had already achieved their best results and were not influenced by further modulation. This could potentially result in a ceiling effect. Studies involving patients with abnormal activity in the noradrenergic system, for instance Parkinson’s disease^64^ or depression^65^ could help to shed light on this, as they may not experience a ceiling effect.

Additionally, we hypothesized that atVNS would only affect trials with active stimulation, rather than during stimulation pauses in the verum condition. This is because previous studies have shown the short-term effects of active stimulation^24,25,27,28,36^. However, contrary to our hypothesis, our results revealed comparable EEG differences between the verum and sham stimulation conditions during the stimulation pause. It was inferred from the pupil reaction that phasic stimulation only lasts for a short time in humans^24,25^, which may not be the best way to determine this. For example, Villani et al. found no carryover effect after three seconds of stimulation on the next trial, nine seconds after the end of the last stimulation^28^. For longer stimulation (30 s), there were signs of changes in markers for tonic activation (higher sAA concentration)^63^. Measured directly in rats, there appears to be a rapid response to the onset and offset of longer stimulation, with reduced activity in the locus coeruleus (LC) during stimulation pauses (30 s ON, 30 s OFF)^36^. Interestingly, prolonged stimulation also leads to a change in the phasic response. Skora et al. showed that 1 s and 30 s stimulation lead to brief pupil dilation and causes phasic activity^66^. Ashton-Jones and Cohen argued that phasic and tonic activity are not two distinct phenomena, but rather that they may be related to each other in an inverted U-shaped manner^19^. Differences in tonic activity lead to differences in the acute phasic response^19^. Although our study revealed differences after stimulus presentation that could be attributed to the phasic response, it remains unclear whether these differences are due to underlying changes in tonic activity. Future studies could address this by evaluating whether short stimulation affects the tonic system.

Apart from the effect of LC activity, the ongoing effect could also be due to sensory factors. Ludwig et al. demonstrated the significance of sensory effects on pupil dilation during atVNS^67^. Our participants were aware of the timing of the stimulation, which therefore might result in expectancy effects.

In conclusion, this study demonstrates that short-lasting, event-related atVNS has an effect on EEG activities, but not on behavioral outcomes during a Go/Nogo task that requires event-file reconfiguration. As EEG changes were detected also in non-stimulated trials, there may be effects of the short-duration atVNS outlasting the period of stimulation.

## Supplementary Information

Below is the link to the electronic supplementary material.

Supplementary Material 1

## Data Availability

The datasets generated during and analyzed during the current study are available from the corresponding author on reasonable request.
