# Supplementary material for: Modulation of response inhibition with event-related atVNS showed EEG but no behavioral effects
Source: Sci Rep. 2025 Nov 19;15:40734. doi: 10.1038/s41598-025-24491-w (PMC12630591; doi:10.1038/s41598-025-24491-w)
Supplement: Supplementary file 1 — Supplementary Material 1 [file 41598_2025_24491_MOESM1_ESM.pdf]

Modulation of response inhibition with event-related atVNS showed EEG but no behavioral effects

Leonie F. Becker[1,2#], Gesine M. Sallandt[1,3#], Eva Rosolowsky[1], Sophie Hetzel[1], Christian Frings[4], Tobias Bäumer[1], Moritz Mückschel[5,6], Christian Beste[5,6,7#*], Alexander Münchau[1#]

Supplementary Information

Tab. S1: Number of trials for MVPA verum against sham for overlapping (o) and non-overlapping (n) trials before averaging. The number of trials used for undersampling is reported.

	Go								Nogo							
Stimulation	Stim		NoStim1		NoStim2		NoStim3		Stim		NoStim1		NoStim2		NoStim3	
Trialtype	o	n	o	n	o	n	o	n	o	n	o	n	o	n	o	n
Mean number of trials	158.7	82.8	159.5	82.4	160.1	83.7	157.7	83.1	84.1	51.0	88.1	50.7	88.2	51.4	88.2	51.9
Minimum number of trials	24	23	57	28	79	53	50	27	23	14	34	16	30	27	33	24
Maximum number of trials	180	90	195	99	197	103	190	106	106	54	122	67	119	67	120	65

Tab. S2: Number of trials for MVPA for stimulated (Stim) against non-stimulated verum trials (NoStim1/NoStim2/NoStim3), separately for overlapping (o) and non-overlapping (n) trials before averaging. The number of trials used for undersampling is reported.

	Go						Nogo					
Stimulation	Stim_NoStim1		Stim_NoStim2		Stim_NoStim3		Stim_NoStim1		Stim_NoStim2		Stim_NoStim3	
Trialtype	o	n	o	n	o	n	o	n	o	n	o	n
Mean number of trials	156.8	81.4	157.8	82.3	155.4	82.0	86.1	50.2	86.8	49.8	85.5	51.3
Minimum number of trials	24	23	24	23	24	23	23	14	23	14	23	14
Maximum number of trials	192	99	193	99	186	106	115	67	119	63	112	65
